# Cardio-Metabolic and Mental Health Outcomes Before and During the COVID-19 Pandemic in a Cohort of Women With Gestational Diabetes Mellitus in Switzerland

**DOI:** 10.3389/fendo.2022.948716

**Published:** 2022-07-22

**Authors:** Dan Yedu Quansah, Leah Gilbert, Christophe Kosinski, Olivier Le Dizès, Antje Horsch, Katrien Benhalima, Emmanuel Cosson, Jardena J. Puder

**Affiliations:** ^1^ Obstetric Service, Department Woman-Mother-Child, Lausanne University Hospital, Lausanne, Switzerland; ^2^ Service of Endocrinology, Diabetes and Metabolism, Department of Medicine, Lausanne University Hospital, Lausanne, Switzerland; ^3^ Institute of Higher Education and Research in Healthcare (IUFRS), University of Lausanne, Lausanne, Switzerland; ^4^ Neonatology Service, Department Woman-Mother-Child, Lausanne University Hospital, Lausanne, Switzerland; ^5^ Department of Endocrinology, UZ Gasthuisberg, KU Leuven, Leuven, Belgium; ^6^ Department of Endocrinology-Diabetology-Nutrition, AP-HP, Avicenne Hospital, Paris 13 University, Sorbonne Paris Cité, CRNH-IdF, CINFO, Bobigny, France; ^7^ Sorbonne Paris Cité, UMR U1153 Inserm/U1125 Inra/Cnam/Université Paris 13, Bobigny, France

**Keywords:** COVID-19, pandemic, gestational diabetes, depression, postpartum, cardio-metabolic, obstetric, neonatal

## Abstract

**Background:**

The COVID-19 pandemic has been associated with worsened metabolic and mental health in the general and perinatal population. The postpartum is a critical moment regarding these outcomes particularly in women with gestational diabetes mellitus (GDM). We investigated the cardio-metabolic and mental health outcomes before and during the pandemic in this population.

**Methods:**

This cohort study included 418 women with GDM, recruited during two distinct periods. This included 180 women exposed to the pandemic (E+) and recruited between May 2020-April 2021 and 238 women who were not exposed to the pandemic during their postpartum period (attended a year before=non-exposed (E-)) and recruited between January-December 2019. Among the E+, a nested-subcohort of 120 women were exposed both during pregnancy and postpartum. During the pandemic, we adopted a hybrid follow-up of women that consisted of in-person consultations, regular contact *via* phone calls (35%), sent recorded exercise guide to patients to follow at home and linked to our website. We specifically focused on maintaining motivation and keeping a strong focus on healthy lifestyle behaviors. Obstetric, neonatal, cardio-metabolic and mental health outcomes were assessed during pregnancy and postpartum.

**Results:**

The pandemic was not associated with worsened weight, weight retention, glucose tolerance, metabolic syndrome, well-being or depression in the postpartum with the exception of a minimally increased HbA1c, diastolic blood pressure and lower emotional eating scores in E+ women (all p ≤ 0.046). In the nested subcohort, E+ women had a slightly increased HbA1c at the first GDM visit and a higher need for glucose-lowering medication (both p ≤ 0.014), but HbA1c at the end of pregnancy and other cardio-metabolic, mental health, obstetric and neonatal outcomes during pregnancy were similar.

**Conclusions:**

The pandemic was not associated with any clinically relevant worsening of cardio-metabolic, mental health, obstetrical and neonatal outcomes in our GDM cohort. This was possibly due to a continued hybrid follow-up, and the partial lockdown in Switzerland.

## Introduction

The world health organization declared Coronavirus disease (COVID-19) a pandemic in March 2020 (1, 2). Following this, countries imposed lockdowns or confinements as measures to contain and prevent the spread of the virus (3). As in other countries, lockdown conditions including contact restrictions, quarantine, and school and company closures changed frequently in Switzerland, but overall, there was mostly a partial lockdown (4–6). This was associated with increased stress, anxiety, depression, lack of exercise and frequent snacking in the population (4–6). An average of 3kg increase in weight during the 2-year pandemic in all age groups and of 6.7kg in middle-aged adults has been reported in Switzerland (7). Similarly, cross-sectional analyses observed an increase in mental health problems in young adults (8) as well as in pregnant and breastfeeding women in Switzerland (9).

Regarding the clinical population, lockdown has been internationally associated with difficulties in diabetes management (10) and an increase in adverse metabolic outcomes including increased body fat, visceral fat, glucose and blood pressure (11, 12). Symptoms of stress, depression, and anxiety also increased in patients with diabetes, whilst well-being decreased (6, 13–15). The lockdown also led to difficulties in the clinical management of antenatal care (9, 16) as online consultations were encouraged to manage specific subgroups, such as women with gestational diabetes mellitus (GDM) (17). In the overall pregnant population, the pandemic led to an increase in adverse obstetric, neonatal, and mental health outcomes (18, 19).

The prevalence of GDM increased during the pandemic (20). Only few data exist regarding the impact of COVID-19 on outcomes of women with GDM. In contrast to the general pregnant population, the pandemic did not lead to an increase in perinatal complications in women with GDM (21, 22). One recent qualitative study that performed phone interviews in 11 women with previous GDM during the pandemic reported negative feelings of loneliness, breastfeeding uncertainties and worries about the infant’s well-being and the risk of type-2 diabetes (16). There are no quantitative studies regarding their mental health outcomes. Data about glycemic control in GDM is conflicting: while one retrospective study found worsened glucose control during pregnancy (23), another did not (21).

The postpartum period is a critical moment regarding weight retention, glucose tolerance and mental health, specifically depression. Weight retention is the most important predictor for future diabetes in GDM women (24). Women with prior GDM also have a 4-fold increased prevalence of postpartum depression compared to women without previous GDM in the postpartum (25). So far, no study has investigated the postpartum outcomes in women with GDM during the pandemic. Knowledge of the metabolic and mental health outcomes in the entire perinatal period during the pandemic could help to guide clinical management in this population. This is especially relevant in view of their increased risk of diabetes, cardiovascular diseases (26, 27) and adverse mental health outcomes.

The aims of this study were to investigate the differences in metabolic, cardiovascular and mental health outcomes in the early postpartum period in a cohort of women with GDM in exposed and non-exposed to COVID-19 pandemic in Switzerland. We also investigated the same outcomes as well as obstetric and neonatal outcomes during pregnancy in a nested subcohort that was exposed to the pandemic during both pregnancy and postpartum.

## Methods

### Study Design and Patient Population

Details regarding this longitudinal clinical cohort following women with GDM during pregnancy and in the postpartum have been previously described (28–32). This cohort investigates lifestyle factors and metabolic consequences of GDM on maternal health and the mother-child relationship during pregnancy up to three years postpartum. The cohort registry started in 2011. The Human Research Ethics Committee of the Canton de Vaud (326/15) approved the study protocol.

For the current study, the inclusion criteria were all women with GDM attending antenatal diabetes care at the Lausanne University Hospital between 2019-2021 and were followed-up during two specific time-periods. First, women who attended their 6-8 weeks postpartum visit during the partial lockdown from May 2020-April 2021 were included as exposed cases (E+), i.e. women whose postpartum visit started at least 2-months after the first COVID-19 wave and lockdown. Part of these women were included in a subcohort because they were exposed to the pandemic both during pregnancy and in the postpartum and had their first visit in the GDM clinic after May 2020. Switzerland experienced two large waves of the pandemic (first wave; 28.02-30.05.2020, second wave; 29.07.2020-17.02.2021) during the study period. Second, women who were followed-up 12-months before the COVID-19 pandemic, i.e. women who had their 6-8 weeks postpartum visit between January and December 2019, were included as non-exposed controls (E-). We did this because we were specifically interested in their health outcomes in the early postpartum period before and during the pandemic.

During these two specific time-periods, 596 women were followed-up in our clinic and had postpartum data. We excluded those with type 1 or type 2 diabetes (n=17). Out of this eligible population (n=579), 161 women did not provide written consent, mostly due to non-attendance of the postpartum visit due to COVID-19 restrictions (27%). Overall, 418 eligible women were included in the final analysis, consisting of 180 E+ (includes 120 women in the nested subcohort) and 238 E- women.

### GDM Diagnosis, Treatment and Patient Follow-up

All women were diagnosed with GDM during pregnancy at 24-28 weeks gestational age (GA), in accordance with the International Association of Diabetes and Pregnancy Study Groups (IADPSG) and the American Diabetes Association (ADA) guidelines (33, 34). Before the partial lockdown restrictions, we routinely followed-up women with GDM clinically according to the current international guidelines (33, 35). They had continuous regular face-to-face appointments every 1-3 weeks with a medical doctor, a diabetes-specialist nurse and/or a dietician after the GDM diagnosis. During these visits, women received information on GDM, specific recommendations regarding lifestyle changes and gestational weight gain (GWG) based on the 2009 recommendations of the Institute of Medicine (IOM) (36). Physical activity was encouraged and counselling with a physiotherapist and/or participation in GDM physical activity groups was proposed. Treatment with insulin or rarely with metformin, was introduced when glucose values remained above targets between two or more times during a 1 to 2-week period despite lifestyle changes in line with Swiss guidelines (37). The 6-8 weeks postpartum visit included a 75g oral glucose tolerance test (OGTT), physical examination and in-person consultation to discuss the postpartum diagnosis and provide counseling. Prior to the pandemic and its associated restrictions, all consultations were in-person.

Regarding the clinical care during the lockdown period, all women had their first GDM visit in-person at the GDM clinic and were taught how to perform capillary glucose measures. Following this, 27% of the follow-up visits during pregnancy were performed *via* teleconsultation as part of newly implemented institutional policy to limit the spread of COVID-19. In our cohort, 26% (108/418) did not speak French and so we particularly tried to maintain face-to-face visits with the help of an interpreter for these women. At 6-8 weeks postpartum, women came to the GDM clinic to undergo OGTT and physical examination. Overall, 35% of them had their clinical visit *via* teleconsultation. We placed a strong focus on lifestyle and behavioral changes and provided patients with home exercise counseling. We sent recorded pregnancy exercises for women to follow (our website: https://www.chuv.ch/fr/dfme/dfme-home/femme-mere/grossesse-accouchement/consultations-dobstetrique/diabete-gestationnel).

### Measures

#### Sociodemographic Variables

Data on maternal socio-demographic characteristics including age, nationality/ethnic origin and educational level were collected during the first GDM visit. We extracted information on previous history of GDM, family history of diabetes, gravida, parity and family social support during pregnancy (living with partner or with support, yes/no) from participants’ medical charts. Pre-pregnancy weight was extracted from participants’ medical charts or, if missing, was self-reported. About 8-10% of pre-pregnancy weight were self-reported. However, we found a high correlation between self-reported pre-pregnancy weight and measured weight.

#### Metabolic and Cardiovascular Health Outcomes

We measured height and weight at the first and last GDM visit, and at 6-8 weeks postpartum to the nearest 0.1 cm and 0.1 kg, respectively, with electronic scales (Seca^®^). BMI was expressed as a ratio of weight in kilograms to the square of height in meters (kg/m^2^). Gestational weight gain (GWG) was defined as the difference in pre-pregnancy weight and weight at the end of pregnancy and weight retention as the difference in pre-pregnancy weight and weight at 6-8 weeks. Excessive GWG was calculated according to IOM 2009 GWG recommendations based on pre-pregnancy BMI (36). Waist circumference was measured with a tape measure and blood pressure with a digital blood pressure monitor (OMRON^®^ HEM-907) during pregnancy and in the postpartum. Data on the need for glucose-lowering medical treatment during pregnancy (use of insulin and/or metformin; yes/no) were extracted from maternal medical records.

HbA1c was assessed using a chemical photometric method (conjugation with boronate; Afinion^®^) at the first GDM visit and with a High Performance Liquid Chromatography method (HPLC) at 6-8 weeks postpartum, according to international guidelines (38). At 6-8 weeks postpartum, we also performed a 2-hr 75-g OGTT and measured fasting lipids (total cholesterol, triglycerides, LDL-and HDL-cholesterol). In the postpartum, we defined prediabetes [fasting plasma glucose (FPG) 5.6-6.9 mmol/l or HbA1c 5.7-6.4% or 2-hr plasma glucose 7.8-11.0 mmol/l] and diabetes (FPG ≥7.0 mmol/l, 2-hr glucose ≥11.1 mmol/l or HbA1c ≥6.5%) according to the ADA criteria (33). The presence of metabolic syndrome (MetS) in the postpartum was defined according to the International Diabetes Federation guidelines (39).

#### Obstetric and Neonatal Outcomes

Obstetric outcomes included gestational hypertension, pre-eclampsia, GA at delivery (weeks), preterm delivery (<37 GA) and caesarean delivery. Neonatal adverse outcomes included macrosomia (birthweight ≥4000g), large-for-gestational-age (LGA) and small-for-gestational-age (SGA) infants. LGA and SGA were defined as sex-and GA-specific birth weight >90th and <10th centile, respectively, according to the International Fetal and Newborn Growth Consortium for the 21st Century (INTERGROWTH-21st) guidelines (40).

#### Mental Health Outcomes

Maternal mental health outcomes included maternal depressive symptoms, well-being and intuitive eating at the first GDM visit and at 6-8 weeks postpartum in all women. We used the Edinburgh Postnatal Depression Scale (EPDS) (validated 10-item questionnaire) to measure maternal depressive symptoms (41). The EPDS has possible scores of 0-30 points; a higher total score indicates more severe depressive symptoms. Maternal well-being was assessed with the WHO-Five Well-Being Index (42), a validated 5-item self-report questionnaire assessed on a 5-point Likert scale ranging from 0 ‘*at no time*’ to 5 ‘*all of the time*’. Total score of the five-items is then multiplied by four (4) to obtain a final score. Possible scores range from 0-100, and higher scores reflect higher well-being status. We assessed intuitive eating with a 14-item self-report questionnaire consisting of “eating for physical rather than emotional reasons” (EPR, 8 items) and the “reliance on hunger and satiety cues” (RHSC, 6 items) subscales of the French Intuitive Eating Scale-2 (IES-2) (43). Scores of IES-2 range between 1 and 5 for each subscale. A higher score of the EPR subscale reflects eating as an answer to hunger and a lower score means eating to cope with emotional distress, whereas a higher score of the RHSC subscale signifies trust in internal cues, and a lower score reflects less ability to regulate food intake.

### Statistical Analysis

We performed all statistical analyses with Stata/SE 15.1 (StataCorp LLC, TX, USA) (44). Demographic and other descriptive variables were presented as means (± standard deviation) or percentages (%) where appropriate. All outcome variables (obstetric and neonatal, metabolic and cardiovascular health, and mental health variables) were normally distributed. We used t-tests (continuous variables) and/or Chi-square (categorical variables) tests to determine and compare the differences in maternal metabolic, cardiovascular, mental health, obstetric and neonatal (the latter two only in the nested subcohort) outcomes during pregnancy and/or at the 6-8 weeks postpartum between women exposed (E*+;* n=418) and non-exposed (E*-*; n=238) to the pandemic. We compared health outcomes between the nested subcohort (n=120/418) of the E+ women who were exposed to the pandemic both in pregnancy and the postpartum with the E- women. We also compared the differences in the change in HbA1c, depression scores, eating scores between pregnancy (first GDM visit) and postpartum period (or end of pregnancy) in E+ and E- women. All statistical significances were two sided and accepted at p<0.05.

## Results

Among the included participants (n=418), 180 (43%) were exposed to the pandemic (E+) during the postpartum and 238 (57%) were non-exposed to the pandemic (E-). Of the 180 E+ women, a nested subcohort of 120 women (67%) was exposed to the pandemic during pregnancy.

Overall, mean age, GA at GDM diagnosis, pre-pregnancy BMI, fasting glucose at GDM diagnosis and weight at the first GDM visit and other socio-demographic and medical characteristics were similar in E+ and E- women (all p>0.05) (**Table 1**).

**Table 1:** Sociodemographic and medical characteristics of participants a year before and during the COVID-19 pandemic.

	COVID-19 pandemic^a^		
Variable	Non-exposed (n=238)	Exposed (n=180)	P-value
	N (%)	N (%)	
Age (years)	33.40 (5.94)	33.51 (5.33)	0.853
GA at first GDM visit (weeks)	28.48 (4.21)	28.56 (2.80)	0.842
Pre-pregnancy BMI (Kg/m^2^)	26.29 ± 5.61	26.85 ± 6.12	0.341
Weight at the first GDM visit	80.00 ± 14.98	80.97 ± 15.99	0.540
Fasting glucose at diagnosis (mmol/l)	5.14 ± 0.63	5.16 ± 0.65	0.916
Nationality/ethnic origin			0.289
Switzerland	69 (29.0)	51 (28.3)	
Rest of Europe + North America	89 (37.4)	71 (39.4)	
Africa	39 (16.4)	28 (15.6)	
Asia + Oceania	27 (11.3)	22 (12.2)	
Latin America	7 (2.9)	8 (4.4)	
Others	7 (2.9)	0 (0)	
Educational level^b^			0.149
No formal education	1 (0.6)	5 (4.1)	
Compulsory school achieved	40 (24.0)	21 (17.2)	
High school	15 (9.0)	14 (11.5)	
General and vocational education	40 (24.0)	25 (20.5)	
University	71 (42.5)	57 (46.7)	
Family history of diabetes^c^			
Yes	137 (57.6)	92 (51.1)	0.189
No	101 (42.4)	88 (48.9)	
Previous history of GDM^d^			
No	196 (82.4)	156 (86.7)	0.231
Yes	42 (17.6)	24 (13.3)	
History of macrosomia			
Yes	18 (13.2)	9 (10.0)	0.463
No	118 (86.8)	81 (90.0)	
Gravida			0.651
1	78 (32.8)	58 (32.2)	
2	57 (23.9)	50 (27.8)	
≥ 3	103 (43.3)	72 (40.0)	
Parity^4^			0.571
0	103 (43.3)	90 (50.0)	
1	74 (31.1)	47 (26.1)	
2	34 (14.3)	24 (13.3)	
≥3	27 (11.3)	19 (10.6)	
Breastfeeding at 6-8 weeks postpartum			
No	35 (14.7)	17 (9.4)	0.244
Yes	203 (85.3)	163 (90.6)	
Mode of breastfeeding			0.671
Stopped before pp visit	27 (13.3)	24 (14.7)	
Exclusive	123 (60.6)	101 (62.0)	
Mixed	53 (26.1)	38 (23.3)	

GDM denotes gestational diabetes mellitus; GA denotes gestational age in weeks; SD denotes standard deviation. ^a^Exposed denotes women who had their 6-8 weeks postpartum visit between the defined COVID-19 pandemic period; Non-exposed denotes women who had their 6-8 weeks postpartum visit in the year before the COVID-19 pandemic period (January-December 2019). ^b^91 controls and 58 cases had missing data on education ^c^Family history of diabetes consists of those with first-degree relationship of the participant (e.g. mother, father, brother, sister, daughter, son) ^d^Only for women who had a previous pregnancy. All values are expressed as n, % or mean (and standard deviation). P value were derived from ANOVA tests for continuous variables and Chi-square test for categorical variables.

### Metabolic and Cardiovascular Health Outcomes in the Early Postpartum

**Table 2** compares the maternal metabolic and cardiovascular health outcomes at 6-8 weeks postpartum in E+ and E- women. The timing of the 6-8 weeks postpartum visit did not differ between both groups (6.3 ± 1.5 *vs* 6.1 ± 1.6 weeks, p=0.588). Similarly, there were no differences in weight, BMI or weight retention at 6-8 weeks postpartum between the E+ and E- women (all p≥0.488). Although there were no differences in fasting glucose, 2-hr glucose after OGTT or prevalence of prediabetes and diabetes, HbA1c was minimally higher in the E+ women (5.34 ± 0.41 *vs* 5.26 ± 0.45, p=0.044). However, the change in HbA1c between the first GDM visit during pregnancy and the 6-8 weeks postpartum visit was similar (p=0.808). Regarding the cardiovascular outcomes studied at 6-8 weeks postpartum, only diastolic blood pressure was higher in the E+ women (74 ± 9 *vs* 72 ± 9, p=0.031) compared to the E- women. None of the cardiovascular health outcomes including total cholesterol, HDL, LDL-cholesterol, triglycerides and the prevalence of the MetS differed significantly (all p≥0.137).

**Table 2:** Maternal cardio-metabolic health outcomes in the postpartum a year before and during the pandemic.

	COVID-19 pandemic^a^		
Variable	Non-exposed (n=238)	Exposed (n=180)	P-value
	Mean ± SD	Mean ± SD	
**Metabolic outcomes**			
Timing of 6-8 weeks postpartum testing (weeks)	6.31 ± 1.52	6.13 ± 1.6	0.588
Weight (kg)	74.15 ± 14.92	75.22 ± 15.77	0.488
BMI (kg/m^2^)	27.76 ± 5.42	28.13 ± 5.59	0.513
Weight retention (kg)	3.88 ± 6.14	3.52 ± 5.92	0.561
Fasting glucose (mmol/l)	5.00 ± 0.53	5.07 ± 0.55	0.224
2-hr glucose after OGTT (mmol/l)	5.44 ± 1.66	5.58 ± 1.48	0.376
HbA1c (%)	5.26 ± 0.45	5.34 ± 0.41	**0.044**
ΔHbA1c (%)^b^	0.025 ± 0.40	0.013 ± 0.48	0.808
Glucose tolerance status (n, %)			0.577
Normal	194 (81.5)	141 (78.3)	
Prediabetes	40 (16.8)	38 (21.1)	
Diabetes	4 (1.7)	1 (0.6)	
**Cardiovascular outcomes**			
Total cholesterol (mmol/l)	5.16 ± 0.99	5.18 ± 0.94	0.834
HDL-cholesterol (mmol/l)	1.51 ± 0.48	1.54 ± 0.62	0.570
LDL-cholesterol (mmol/l)	3.09 ± 0.83	3.31 ± 2.66	0.258
Triglycerides (mmol/l)	1.30 ± 0.86	1.45 ± 1.36	0.189
Systolic blood pressure (mmHg)	110.55 ± 11.97	112.35 ± 12.09	0.137
Diastolic blood pressure (mmHg)	72.17 ± 9.37	74.24 ± 9.39	**0.031**
Metabolic Syndrome (n, %)			
Waist circumference-defined	70 (29.4)	49 (27.2)	0.623
BMI-defined	44 (18.4)	23 (12.8)	0.115

HbA1c denotes glycated hemoglobin; BMI denotes body mass index; OGTT denotes 75g oral glucose tolerance test; HDL denotes high-density lipoproteins; LDL denotes low-density lipoproteins.

a Exposed denotes women who had their 6-8 weeks postpartum visit between the defined COVID-19 pandemic period; Non-exposed denotes women who had their 6-8 weeks postpartum visit in the year before the COVID-19 pandemic period (January-December 2019). All values are expressed as mean and standard deviation unless otherwise stated.

b ΔHbA1c denotes the change in HbA1c between the first GDM visit and 6-8 weeks postpartum.

P value were derived from ANOVA tests for continuous variables and Chi-square test for categorical variables. Bold p values are significant p < 0.05.

### Maternal Mental Health Outcomes in the Early Postpartum

**Table 3** shows the mental health outcomes at 6-8 weeks postpartum in the E+ and E- women. Well-being, overall depression symptom scores, the prevalence of moderate or elevated symptom scores or intuitive eating assessment (hunger and satiety feelings; RHSC subscale) did not differ between both groups (all p≥0.153) except for lower emotional eating (EPR subscale) scores (3.68 ± 0.75 *vs* 4.12 ± 0.78, p=0.046). However, the improvement in emotional eating scores between the first GDM visit during pregnancy and the 6-8 weeks postpartum visit was similar (p=0.754).

**Table 3:** Maternal mental health outcomes in the postpartum a year before and during the pandemic.

	COVID-19 pandemic^a^		
Variable	Non-exposed (n=238)	Exposed (n=180)	P-value
	Mean ± SD	Mean ± SD	
Well-being score at the 6-8 weeks pp visit^b^	63.16 ± 19.42	65.73 ± 19.05	0.302
Total EPDS score at the 6-8 weeks pp visit^c^	6.02 ± 4.10	6.36 ± 5.31	0.153
Symptoms of depression (EPDS)			0.817
Minimal (EPDS <10) (n, %)	204 (85.7)	146 (81.1)	
Moderate (EPDS ≥10-12) (n, %)	12 (5.0)	10 (5.6)	
Elevated (≥13) (n, %)	22 (9.3)	24 (13.3)	
EPR score at the 6-8 weeks pp visit^d^	4.12 ± 0.78	3.68 ± 0.75	**0.046**
ΔEPR^e^	0.23 ± 0.43	0.18 ± 0.57	0.754
RHSC score at the 6-8 weeks pp visit^f^	3.45 ± 1.0	3.48 ± 0.98	0.957
Social support during pregnancy^g^			
Yes	215 (90.3)	154 (85.6)	0.132
No	23 (9.7)	26 (14.4)	

a Exposed denotes women who had their 6-8 weeks postpartum visit between the defined COVID-19 pandemic period; Non-exposed denotes women who had their 6-8 weeks postpartum visit in the year before the COVID-19 pandemic period (January-December 2019).

b The World Health Organization-Five Well-Being Index (WHO-5) (a higher score reflects higher well-being status and a lower score indicates lower well-being).

c EPDS denotes Edinburg Postnatal Depression Scale (a higher total score indicates more severe depressive symptoms).

d EPR denotes eating for physical rather than emotions subscale of the Intuitive Eating Scale-2 (IES-2) (a higher score reflects eating as an answer to hunger and a lower score means eating to cope with emotional distress).

e ΔEPR denotes the change in EPR between the first GDM visit and 6-8 weeks pp.

f RHSC denotes reliance on hunger and satiety cues subscale of the Intuitive Eating Scale-2 (IES-2) (a higher score signifies trust in internal cues, and a lower score reflects less ability to regulate food intake).

g Yes denotes women who lives with their partner or who live alone with support.

All values are expressed as mean and standard deviation. P value was derived from ANOVA test. Bold p values are significant (p<0.05).

### Metabolic, Cardiovascular, Obstetric, Neonatal and Mental Health Outcomes During Pregnancy

**Table 4** compares the metabolic, cardiovascular, obstetric and neonatal outcomes in the 120 women exposed to the pandemic both during pregnancy and in the postpartum (nested subcohort) and the E- women. E+ women had an increased HbA1c at the first GDM visit (5.36 ± 0.37 *vs* 5.25 ± 0.36, p=0.014) and a higher need for glucose-lowering medication (62% *vs* 45%, p<0.001) during pregnancy. However, HbA1c at the end of pregnancy and the change in HbA1c between the first GDM visit and the end of pregnancy were not different (both p≥0.155). Other cardio-metabolic outcomes studied during pregnancy did not differ (all p ≥0.155). Obstetric and neonatal outcomes including LGA, SGA, macrosomia and caesarean delivery were also similar (all p ≥0.176). There were no significant differences in maternal well-being, overall depression symptom scores or the prevalence of moderate or elevated symptom scores or intuitive eating scores during pregnancy between E+ and E- women (all p ≥0.352) (**Table 5**). Family social support during pregnancy did not differ (p=0.143) between groups.

**Table 4:** Maternal metabolic, cardiovascular, obstetric and neonatal health outcomes during pregnancy a year before and during the pandemic. .

	COVID-19 pandemic^a^		
Variable	Non-exposed (n=238)	Exposed (n=120)	P-value
	Mean ± SD	Mean ± SD	
**Before and during pregnancy**			
Pre-pregnancy weight (kg)	70.30 ± 15.72	72.72 ± 16.88	0.169
Pre-pregnancy BMI (Kg/m^2^)	26.19 ± 5.66	27.29 ± 6.16	0.084
Weight at the first GDM visit	79.67 ± 15.23	82.08 ± 15.73	0.154
1hr glucose at GDM diagnosis (mmol/l)	9.44 ± 1.83	9.60 ± 1.74	0.513
2hr glucose at GDM diagnosis (mmol/l)	7.73 ± 1.81	7.93 ± 1.92	0.410
HbA1c at the first GDM visit (%)	5.25 ± 0.36	5.36 ± 0.37	**0.014**
Need for glucose-lowering treatment (yes) (n, %)	108 (45.4)	74 (61.7)	**<0.001**
SBP at the first GDM visit (mmHg)	109.95 ± 11.41	110.0 ± 12.41	0.972
DBP at the first GDM visit (mmHg)	70.28 ± 9.63	70.51 ± 9.73	0.835
**At the end of pregnancy**			
Gestational weight gain (kg)	12.08 ± 6.79	11.27 ± 7.42	0.337
IOM weight gain recommendation (n, %)			0.319
Below recommendation	87 (36.5)	46 (38.3)	
Within recommendation	82 (34.5)	35 (29.2)	
Above recommendation	69 (29.0)	39 (32.5)	
HbA1c at the end of pregnancy (%)	5.34 ± 0.38	5.41 ± 0.45	0.155
ΔHbA1c during pregnancy^b^	0.09 ± 0.24	0.05 ± 0.45	0.497
SBP at the end of pregnancy (mmHg)	112.20 ± 10.80	113.17 ± 11.21	0.451
DBP at the end of pregnancy (mmHg)	73.18 ± 9.81	74.72 ± 10.23	0.187
**Obstetric and neonatal outcomes**			
Gestational hypertension (yes) (n, %)	5 (2.1)	0 (0)	0.147
Pre-eclampsia (yes) (n, %)	8 (3.4)	4 (3.3)	0.776
Gestational age at delivery (weeks)	38.86 ± 1.91	39.03 ± 5.47	0.643
Caesarean delivery (yes) (n, %)	104 (43.7)	38 (31.7)	0.528
Prematurity, yes (%)^c^	23 (9.7)	13 (10.8)	0.480
Birth weight (g)	3240.52 ± 74	3239.82 ± 61	0.998
Macrosomia (yes) (%)^d^	35 (14.7)	9 (7.5)	0.176
Large-for-gestational-infant, yes (%)	45 (18.9)	15 (12.5)	0.316
Small-for-gestational-age infant, yes (%)	35 (14.7)	16 (13.3)	0.891

HbA1c denotes glycated hemoglobin; BMI denotes body mass index; SBP denotes systolic blood pressure; DBP denotes diastolic blood pressure; IOM denotes Institute of Medicine gestational weight gain recommendation 2009.

a Exposed denotes women who had their 6-8 weeks postpartum visit between the defined COVID-19 pandemic period; Non-exposed denotes women who had their 6-8 weeks postpartum visit in the year before the COVID-19 pandemic period (January-December 2019).

b Difference between HbA1c at the end of pregnancy and at the first GDM visit.

c Prematurity defined at infant delivery <37 gestational age.

d Macrosomia defined as birthweight ≥4000g.

All values are expressed as mean and standard deviations unless otherwise stated. P value derived from ANOVA test for continuous variables and Chi-square test for categorical variables. Bold p values are significant (p<0.05).

**Table 5:** Maternal mental health outcomes during pregnancy a year before and during the pandemic.

Variable	COVID-19 pandemic^a^		P-value
	Non-exposed (n=238)	Exposed (n=120)	
	Mean ± SD	Mean ± SD	
Well-being score at the first GDM visit^b^	59.06 ± 20.69	61.76 ± 20.20	0.258
Total EPDS score at the first GDM visit^c^	7.47 ± 5.43	7.63 ± 5.39	0.802
Symptoms of depression (EPDS)			0.352
Minimal (EPDS <10) (n, %)	152 (72.4)	71 (68.3)	
Moderate (EPDS ≥10-12) (n, %)	19 (9.0)	15 (14.4)	
Elevated (≥13) (n, %)	39 (18.6)	18 (17.3)	
EPR score at the first GDM visit^d^	3.78 ± 0.83	3.86 ± 0.84	0.590
RHSC score at the first GDM visit^e^	3.62 ± 0.81	3.63 ± 0.79	0.986
Social support during pregnancy^f^			
Yes	194 (81.5)	111 (92.5)	0.143
No	44 (18.5)	9 (7.5)	

a Exposed denotes women who had their 6-8 weeks postpartum visit between the defined COVID-19 pandemic period; Non-exposed denotes women who had their 6-8 weeks postpartum visit in the year before the COVID-19 pandemic period (January-December 2019).

b The World Health Organization-Five Well-Being Index (WHO-5) (a higher score reflects higher well-being status and a lower score indicates lower well-being).

c EPDS denotes Edinburg Postnatal Depression Scale (a higher total score indicates more severe depressive symptoms).

d EPR denotes eating for physical rather than emotions subscale of the Intuitive Eating Scale-2 (IES-2) (a higher score reflects eating as an answer to hunger and a lower score means eating to cope with emotional distress).

e RHSC denotes reliance on hunger and satiety cues subscale of the Intuitive Eating Scale-2 (IES-2) (a higher score signifies trust in internal cues, and a lower score reflects less ability to regulate food intake).

f Yes denotes women who lives with their partner or who live alone with support.

All values are expressed as mean and standard deviation. P value was derived from ANOVA test.

## Discussion

In this clinical cohort of women with GDM, there were no clinically relevant worsening of weight, weight retention, glucose tolerance, metabolic syndrome, well-being, depression or other cardio-metabolic and mental health outcomes in the early postpartum between women exposed (E+) and non-exposed (E-) to the pandemic. Exceptions were a small increase in HbA1c and diastolic blood pressure and lower emotional eating scores in E+ women than in E-women. In a nested subcohort that was also exposed to the pandemic during pregnancy, E+ women had a slightly higher HbA1c at the first GDM visit, but not at the end of pregnancy, and a higher need for glucose-lowering medication. However, other cardio-metabolic, mental health, obstetric and neonatal outcomes during pregnancy or at birth did not differ.

Between May 2020 and April 2021 when this study was conducted, Switzerland experienced two different waves of the pandemic. Compared to total lockdown restrictions in other European countries, Switzerland imposed less stringent, mostly partial lockdown measures, and changed frequently. This led to an overall increase in weight and mental health problems in the general Swiss population (7–9), and these adverse outcomes have also been reported globally outside the perinatal period (18, 19). Women with GDM have an increased risk for adverse maternal metabolic, cardiovascular and mental health outcomes compared to the general population. The postpartum period is a critical moment for women with GDM, especially regarding their increased risk of depression, future diabetes and cardiovascular disease (26, 27). Knowledge of these outcomes in the entire perinatal period during the pandemic could help to guide clinical management.

The 0.08 ± 0.42 mmol/l increase in HbA1c in E+ women compared to E-women in the postpartum might be due to the modified dietary habits such as frequent snacking reported in the pandemic (4–6), but this appears not clinically relevant. In addition, there were no differences in the changes in HbA1c between the first GDM and the postpartum visit and other metabolic outcomes in the postpartum including weight and weight retention. Glucose tolerance status, lipid levels and the prevalence of the MetS were also not different. However, we observed a 2.1 ± 9.0 mmHg increase in diastolic, but not systolic blood pressure in E+ women than in E- women. Although such an increase can be relevant on a population level, it is too small for an individual impact on cardiovascular risk, especially at observed low blood pressures (systolic blood pressure <140 mmHg) (45, 46). Moreover, the difference in diastolic blood pressure was no longer significant after adjusting for weight (data not shown) and thus may be related to weight and/or be multifactorial including changes in physical activity, eating habits or stress, for which we did not have detailed measures. Taken together, these findings are very encouraging, especially when considering the significant 3-6 kg increase in weight in the general population in Switzerland and the increase in GDM prevalence observed in several countries (7). The type or nature of lockdown restrictions (partial) in Switzerland, but particularly the continued GDM follow-up consultations during the pandemic with a focus on healthy lifestyle might explain the lack of differences in clinically relevant outcomes in E+ and E- women as opposed to the general population.

Regarding mental health, the pandemic was not associated with increased depression scores or reduced well-being in the early postpartum. This contradicts the increased stress, depression, well-being and anxiety symptoms (6, 13–15) reported in the general non-pregnant population and in pregnant and breastfeeding women during the pandemic (9). In the general pregnant population, fear of infection or death, and disruption of human relationships during the pandemic correlated with depression symptoms and well-being (47). As women with GDM have an increased risks of depression in the perinatal period compared to the healthy pregnant population (25), it is surprising that the pandemic did not similarly exacerbate these risks in our cohort. The continuous medical social support may have alleviated the burden of the lockdown, but further investigation is required. On the other hand, E+ women in our cohort had about 10% lower emotional eating scores in the early postpartum compared to E- women. This lower score suggest that E+ women were more frequently eating to cope with emotional distress. This could be explained by the increased stress levels and frequent snacking reported in the general population during the lockdown and might be a specific expression of reduced mental health (6, 13–15). Importantly, this difference in emotional eating did not have a significant impact on weight and weight retention or overall mental health in the postpartum period, and its change between pregnancy and the early postpartum was similar in both groups.

In the nested subcohort of women exposed to the pandemic both during and after pregnancy, HbA1c at the first GDM visit, i.e. before the follow-up, and the need for glucose-lowering medication treatment during pregnancy were increased in E+ women. The change in HbA1c between the first GDM visit and the end of pregnancy was not different between groups. Only two studies have investigated glycemic control during pregnancy in the pandemic and the results are controversial. Whereas one showed no differences in glycemic parameters in GDM women before and during the pandemic (21), another study that reported lower glucose control and an increased need for insulin treatment during pregnancy in women with GDM during the pandemic, is consistent with our result (23). Our findings could be explained by the decreased physical activity during the lockdown. Although we placed a focus on continuing home exercise during the lockdown and sent recorded pregnancy exercises for women to follow, the daily commuting to work or school, and the overall general activity was most likely reduced and home exercise was not always feasible.

The increase in adverse obstetric and neonatal outcomes reported in the general pregnant population during the pandemic (18, 19) could be of concern to the GDM population as their risk is already increased (26, 27). However, in our cohort, obstetric and neonatal adverse outcomes including caesarean delivery, prematurity and particularly SGA and LGA potentially related to an increase in stress or decrease in physical activity, were not different. This is in line with two previous studies that did not find worsened perinatal complications in women with GDM during the pandemic (21, 22). Importantly, the pandemic was also not associated with any increase in adverse mental health outcome during pregnancy in our cohort.

The results of this study are encouraging and show that despite the difficulties in a health crisis like the COVID-19 pandemic, GDM follow-up can be adapted in functioning to prevent the worsening of adverse outcomes. We believe that our encouraging results were due to the continued functioning of our GDM clinic during the pandemic and its associated restrictions. We adapted our GDM follow-up to include not only face-to-face consultations, but also frequent phone exchanges, sent recorded exercise proposals to patients to follow at home with their family and linked them to our website. We specifically focused on providing support and maintaining motivation and keeping a strong focus on a healthy lifestyle. This may have helped to alleviate the negative impacts of the pandemic in our patients and positively increased motivation, self-confidence and adherence to comply with lifestyle advice. In future similar pandemic situations, this approach could be used/further adapted to prevent adverse metabolic and mental health outcomes in this population. Our remote follow-up of patients in situations like the pandemic offer a unique advantage to continuously monitor, motivate and provide care to patients despite the restrictions imposed by the pandemic.

This study has several strengths. It is the first to investigate all relevant outcomes of cardio-metabolic and mental health in the entire perinatal period in women with GDM exposed to the pandemic with a focus on the postpartum and included a very similar control group of women with GDM who were not exposed to the pandemic. The ethnically diverse cohort, reflective of the population in Switzerland, also increases the external validity of our results. Despite this, we cannot confirm the causality of our associations. The partial lockdown in Switzerland limits the generalizability of our results to other countries that experienced total lockdown measures. In addition, detailed data on glycemic values and data on nutrition and physical activity were lacking. It is also important to indicate that a quarter (27%) of our eligible population did not consent. This was mostly due to non-attendance at the moment of signing the consent during the postpartum visit in the context of COVID-19 restrictions at times when we restricted postpartum visits due to mobilisation of the clinical team and thus had to focus on providing care during pregnancy. This might influence our results towards reducing any differences in clinically relevant outcomes especially if these patients were high-risk and had adverse outcomes.

## Conclusions

In this clinical cohort of consecutive women with GDM, the COVID-19 pandemic was not associated with any clinically relevant worsening of obstetric, neonatal, metabolic, cardiovascular or mental health outcomes during pregnancy and in the postpartum period. This is in contrast to significant changes in weight and mental health reported by other studies in the general population in Switzerland during the same period. These findings are important in view of the critical postpartum period regarding future diabetes, cardiovascular disease and depression. The partial lockdown in Switzerland together with our continued hybrid follow-up of women with GDM that focused on behavioral and lifestyle changes during the pandemic may explain these encouraging findings.

## Data Availability Statement

The raw data supporting the conclusions of this article will bemade available by the authors, without undue reservation.

## Ethics Statement

The studies involving human participants were reviewed and approved by The Human Research Ethics Committee of the Canton de Vaud (326/15). The patients/participants provided their written informed consent to participate in this study.

## Author Contributions

DQ and JP conceived the study. All authors designed the study. DQ and JP performed the data analysis. All authors were involved in the interpretation of data. DQ and JP wrote the draft manuscript. All authors revised the manuscript for important intellectual content and gave final approval for the version to be published. JP had the idea of the cohort and supervised all the work.

## Funding

This study is a pilot of a project grant by the Swiss National Science Foundation (SNF 32003B_176119). The cohort database received an unrestricted educational grant from Novo Nordisk. The SNF and Novo Nordisk had no role regarding the content of the original data or analyses or in the drafting of this manuscript and did not impose any restrictions regarding the publication of the report.

## Conflict of Interest

The authors declare that the research was conducted in the absence of any commercial or financial relationships that could be construed as a potential conflict of interest.

## Publisher’s Note

All claims expressed in this article are solely those of the authors and do not necessarily represent those of their affiliated organizations, or those of the publisher, the editors and the reviewers. Any product that may be evaluated in this article, or claim that may be made by its manufacturer, is not guaranteed or endorsed by the publisher.

## Abbreviations

ADA, American Diabetes Association; COVID-19, Coronavirus disease ; EPR, Eating for physical rather than emotional reasons; EPDS, Edinburgh Postnatal Depression Scale; E+, Exposed cases; GDM, Gestational diabetes mellitus; GWG, Gestational weight gain; GA, Gestational age; IOM, Institute of Medicine; IADPSG, International Association of Diabetes and Pregnancy Study Groups; IES-2, Intuitive eating scale-2; LGA, Large-for-gestational-age; E-, Non-exposed controls; OGTT, Oral glucose tolerance test; RHSC, Reliance on hunger and satiety cues; SGA, Small-for-gestational-age.
